# An Efficient Improved Harris Hawks Optimizer and Its Application to Form Deviation-Zone Evaluation

**DOI:** 10.3390/s23136046

**Published:** 2023-06-29

**Authors:** Guangshuai Liu, Zuoxin Li, Si Sun, Yuzou Yang, Xurui Li, Wenyu Yi

**Affiliations:** 1 School of Mechanical Engineering, Southwest Jiaotong University, Chengdu 610031, China; lizuoxin@my.swjtu.edu.cn (Z.L.); yuzhouyang@my.swjtu.edu.cn (Y.Y.); xuruili@my.swjtu.edu.cn (X.L.); 2 Institute of Optics and Electronics, Chinese Academy of Sciences, Chengdu 610209, China; sunsi@ioe.ac.cn; 3 Sichuan Research & Design Institute of Agricultural Machinery, Chengdu 610066, China; ywycd@163.com

**Keywords:** Harris hawks optimization, salp swarm algorithm, minimum-zone evaluation, form error, tolerancing

## Abstract

Evaluation of the deviation zone based on discrete measured points is crucial for quality control in manufacturing and metrology. However, deviation-zone evaluation is a highly nonlinear problem that is difficult to solve using traditional numerical optimization methods. Swarm intelligence has many advantages in solving this problem: it produces gradient-free, high-quality solutions and is characterized by its ease of implementation. Therefore, this study applies an improved Harris hawks algorithm (HHO) to tackle the problem. The average fitness is applied to replace the random operator in the exploration phase to solve the problem of conflicting exploration strategies due to randomness. In addition, the salp swarm algorithm (SSA) with a nonlinear inertia weight is embedded into the HHO, such that the superior explorative ability of SSA can fill the gap in the exploration of HHO. Finally, the optimal solution is greedily selected between SSA-based individuals and HHO-based individuals. The effectiveness of the proposed improved HHO optimizer is checked through a comparison with other swarm intelligence methods in typical benchmark problems. Moreover, the experimental results of form deviation-zone evaluation on primitive geometries show that the improved method can accurately solve various form deviations, providing an effective general solution for primitive geometries in the manufacturing and metrology fields.

## 1. Introduction

Primitive geometries are widely used in aerospace, shipbuilding, and medicine. The form deviation of primitive geometries affects part mating. Part mating is critical in manufacturing and metrology quality control, affecting assembly, service life, wear resistance, and motion. Therefore, the development of computational algorithms to improve the efficiency and reliability of the production processes for manufactured parts has been a challenging research task during the last three decades. The rapid acquisition of discrete point clouds from surfaces has become possible with the development of coordinate measuring machines (CMMs) and low-cost 3D acquisition techniques. Therefore, inspecting manufactured parts by coordinate metrology on a discrete point cloud is an effective method for assessing the degree of satisfaction with design requirements.

Typically, the measured points should be compared to the ideal geometry to determine whether the part is to be accepted or rejected [1]. Robust algorithms can quickly determine a substitute geometry in point clouds, but the reliability and efficiency of the algorithms are affected by the inherent uncertainty of the equipment used. Uncertainty may arise either from systematic errors [2] or data noise [3]. Hence, numerous coordinate metrology tasks focus on eliminating the uncertainty associated with data acquisition, which is referred to as point measurement planning (PMP) [4]. Since the measurement time is proportional to the number of points, PMP research focuses on the size and location of the measurement process to achieve a more precise representation of the measured geometries using fewer points [5]. The sampling strategy design is a solution for contact measurements. Sampling strategies in the literature can be divided into three main categories: uniform, random, and stratified sampling [6]. Noncontact measurements provide more surface information [7], but at the same time, the increased density leads to computational instability and cost. Gohari et al. [8] introduced a data-mining algorithm that analyzes the trend of errors for the acquired points, which guarantees a reliable evaluation of geometric and form deviations.

The second computational task, which is also the main focus of this research work, is substitute geometry estimation (SGE). The objective of SGE is to obtain the ideal geometry parameters for the measured points or to locate the ideal geometry for the measured points via different types of fitting criteria, such as least-squares fitting, total least-squares fitting, min–max fitting, and minimum-zone fitting. In the former, fitting occurs directly on the point cloud, and the latter aligns the point cloud with the design model. The least-squares method is widely used in surface error estimation owing to its high evaluation efficiency. However, it does not strictly adhere to the minimum zone specified by ISO [9] and can only provide approximate results, which may lead to misjudgment of the workpiece and economic losses. The minimum-zone method is often used as the basis for arbitration among various evaluation methods since it is more consistent with the standard definition of physical fittings [10]. Nevertheless, minimum-zone deviation is a highly nonlinear problem, and multivariate optimization algorithms are required to provide satisfactory substitute geometries.

There have been many studies that have applied numerical optimization methods to the evaluation of form errors, including simplex search [11,12], semidefinite programming [13], linear approximation [14], iterative reweighted [15], Chebyshev approximation [16], and steepest descent [17]. In the case of higher nonlinearity, it is challenging for these algorithms to obtain the global solution, since several local solutions may exist. Increasing the number of sample points also reduces the chance of obtaining the global minima in the employed optimization process [18]. Based on this, many researchers have developed new data-fitting methods to solve the above problems.

The representation of surfaces by convex hulls is common in computational geometry techniques, and many researchers have applied it to form error evaluations [19,20]. In addition to the convex-hull technique, Liu et al. [21] constructed the minimum-zone roundness intersection structure and evaluation model using the crossing relationship of chords. In a subsequent study [22], the method was expanded to cylindricity evaluation. In addition, based on computational geometry techniques, Alhadi et al. [23] presented an improved algorithm for the minimum zone of roundness error evaluation using an alternating exchange method. A minimum-zone fitting function was created to enhance the roundness error evaluation. Zhuo et al. [24] introduced the definition of the crossing sector structure based on the minimum-zone criterion and transformed it into an angular relationship of control points, making it easy to identify the MZC. For straightness error, Li et al. [25] proposed a simple bidirectional algorithm based on a four-point model for the calculation of the minimum-zone straightness error from planar coordinate data. Four points are used to construct the upper and lower reference lines which can select candidate points effectively by comparing the slope of the upper and lower reference lines. It is worth noting that computational geometry techniques suffer from the inherent problem of poor solution accuracy. Therefore, combining the initial solution and region search algorithm to search the parameter space greedily is a new research direction. Ye et al. [26] proposed a new neighborhood-based adaptive iterative search strategy. The results of the proposed method provide more accurate values than conventional techniques. Huang et al. [27] presented an asymptotic search method according to which roundness is solved iteratively using the intersecting chord to avoid trapping in the local solution. Liu et al. [28] proposed and developed a novel cylindricity evaluation method. The framework and information flow of the algorithm has been documented, together with the description of the six-point subset, the replacement strategy, and the terminal condition. However, the greedy search approach does not guarantee a global solution and is often inefficient when data increase. There is still a demand for a comprehensive and stable method.

In recent studies, researchers have successively adopted swarm intelligence (SI) to resolve these issues. Some well-known SI methods and many improved optimization algorithms have been effectively used to determine substitute geometries according to various criteria. Du [29] and Pathak [30] applied particle swarm algorithms (PSO) to evaluate form error. Zhang et al. [31] applied an ant-colony algorithm (ACO) to straightness, but it easily fell into local optimal solutions, so Luo et al. [32] applied an improved artificial bee-colony (ABC) algorithm to straightness error evaluation; however, there was still a lack of accuracy. Based on this, Luo [33] proposed to use an improved differential evolution algorithm (DE) for straightness evaluation. For roundness, Wen et al. [34] proposed the use of a genetic algorithm (GA) for the evaluation of the minimum-zone circle, but the genetic algorithm requires the adjustment of numerous parameters. Recently, Li et al. [35] proposed an improved bat algorithm (BA) to achieve accurate evaluation of minimum-zone roundness. In addition, the application of a genetic algorithm [10] and an improved cuckoo search (CS) [36] algorithm to flatness has been studied. These advanced optimization algorithms have their own advantages and disadvantages. Genetic algorithms can be applied to various complex optimization problems in reality but need to adjust various operators, such as crossover, mutation, and selection. The particle swarm optimization algorithm still needs to adjust the inertia weights for different problems to avoid falling into a local optimum. The CS algorithm, based on the foraging behavior of cuckoos, can obtain high-quality solutions, but the convergence speed is slow.

Harris hawks optimization (HHO) [37] has received extensive attention from the research community. The construction of HHO mimics the foraging behavior of Harris hawks in nature. HHO is designed with two phases of exploration and four phases of exploitation. The results of testing for benchmark functions and several engineering optimization problems confirm that HHO outperforms many well-known SI approaches, such as PSO, GWO, CS, DE, and WOA. Notably, HHO expresses a highly exploitative ability in later stages. SSA [38] is also a well-established swarm intelligence technique based on the salp chain, which simulates the foraging patterns in oceans. Due to its simplicity and superiority, it has been widely used in unconstrained and constrained optimization problems.

In this paper, an improved HHO algorithm (IHHO) is proposed for solving the form deviation-zone evaluation problem. The IHHO focuses on two areas of improvement: exploration strategy selection and exploration capabilities. The latter was mainly inspired by [39]. Furthermore, the search area of the primitive geometries is analyzed to speed up the convergence.

The rest of the paper is organized as follows: Section 2 presents the modeling of the objective function and the determination of the search area of the primitive geometries. An overview of the optimizer is also described. The specific structure of the proposed optimizer is presented in Section 3. Section 4 describes a group of experiments and analyses of the global benchmark problem. Section 5 verifies the practicality of the proposed optimizer in dealing with the form deviation-zone evaluation problem. Finally, conclusions are drawn in Section 6.

## 2. Materials and Methods

### 2.1. Form Deviation-Zone Evaluation Model

The minimum zone is the basic principle of assessing the form error and is the final basis for arbitration in the event of a dispute. To obtain a reliable form deviation zone, it is necessary to establish an optimization objective function according to the minimum-zone criterion based on the distance function of each primitive geometry. Assuming that $P_{i}\left(x_{i},y_{i},z_{i}\right)$ is a discrete measurement point acquired from the surface, $f(P_{i},U)$ denotes the distance function from the point to the ideal surface, where *U* denotes the fitted parameter; then:

(1) $$e_{i}=\underset{i}{\max}\{f(P_{i},U)\}-\underset{i}{\min}\{f(P_{i},U)\}$$

The objective function satisfying the minimum-zone criterion is:

(2) $$F(P_{i},U)=\min\{e_{i}\}$$

The deviation-zone evaluation process solves Equation (2) by continuously optimizing the fitting parameter, *U*, to minimize the objective function. Obviously, diverse geometries have different expressions and distance functions, and the number of parameters to be optimized varies. In the following, these surfaces will be individually discussed.

1. Roundness

**Figure 1:**
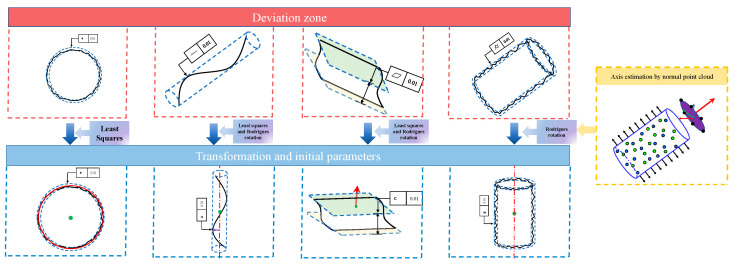
The least-squares method is used to estimate the initial parameters, and the Rodrigues rotation matrix aligns the original geometry with the *Z*-axis.

**Figure 2:**
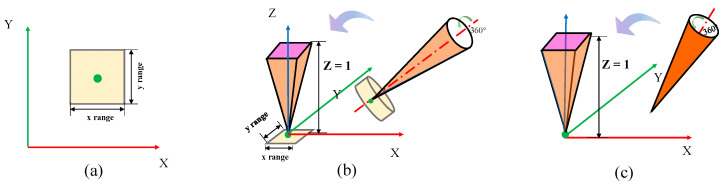
The search area in the optimization process: (**a**) for circles; (**b**) for lines and cylinders, divided into regions of position (yellow) and areas of axial direction (orange); and (**c**) for planes, only areas of direction.

The circle is one of the most common features of industrial annular workpiece parts. Expression equations and distance functions for circles and other geometries are given in Appendix A. Although the distance function is related to the circle center, (a,b), and the radius, *R*, the radius cancels out while subtracting the maximum and minimum distances. Therefore, the variables to be optimized are the circle center coordinates, which is a two-dimensional optimization problem. We determine the center and roundness error by the least-squares method, as shown in Figure 1, and the search area of the proposed optimization algorithm is shown in Figure 2a.

2. Straightness

A spatial line has six parameters: three for position, $\left(x_{0},y_{0},z_{0}\right)$ , and three for direction, (a,b,c). According to error theory, the arithmetic mean of the measurement sequence is the closest to its actual value, so the arithmetic mean of the line is used as the spatial line position. Typically, the minimum-zone line is around the least-squares line. Consequently, the initial linear direction vector is obtained by least squares, as shown in Figure 1. However, the direction of the spatial line is arbitrary, and it is often difficult to average over all directions when determining the search area by the least-squares parameters. With this in mind, we align the line to the *Z*-axis by the Rodrigues rotation matrix, T, to reduce the optimization dimension and determine the appropriate search area. The transformation process is as follows:

(3) $$P_{ij}=TP_{ij}$$

(4) $$T=\left[\begin{array}{ccc} \cos(\theta )+a^{2}(1\;-\;\cos(\theta )) & -c\sin(\theta )+ab(1\;-\;\cos(\theta ))\; & b\sin(\theta )+ac(1\;-\;\cos(\theta ))\\ c\sin(\theta )+ab(1\;-\;\cos(\theta )) & \cos(\theta )+b^{2}(1\;-\;\cos(\theta )) & -a\sin(\theta )+bc(1\;-\;\cos(\theta ))\\ -b\sin(\theta )+ac(1\;-\;\cos(\theta )) & \mathrm{asin}(\theta )+bc(1\;-\;\cos(\theta )) & \cos(\theta )+c^{2}(1\;-\;\cos(\theta )) \end{array}\right]$$

where θ denotes the angle between the line and the *Z*-axis, satisfying the following equation:

(5) $$\cos(\theta )=\frac{n\cdot z}{\left|n\right|\left|z\right|}$$

The search space of the position parameter can then be centered on the projection point of the centroid in the *XY*-plane. The search space of the direction parameter can be centered on the *Z*-axis. Then, the line expression can be simplified as:

(6) $$\frac{X-x_{0}}{p}=\frac{Y-y_{0}}{q}=\frac{Z}{1}$$

Through the above process, the straightness evaluation becomes a four-dimensional optimization problem, and the search area is shown in Figure 1b.

3. Cylindricity

Many parts designed in machines have a cylindrical geometry. Compared to roundness, cylindricity takes into account both axial and radial directions. The distance function of a cylinder is $f(P_{i},x_{0},y_{0},z_{0},a,b,c)$ (given in Appendix A), including the axis position, $\left(x_{0},y_{0},z_{0}\right)$, the axis direction, (a,b,c), and the cylindrical radius, R. Similar to the circle, *R* can be disregarded in deviation-zone evaluation. Therefore, cylindricity evaluation is essentially the determination of the position and direction of the cylindrical axis. It is a six-dimensional optimization problem similar to the spatial line. Thus, the dimension-reduced method of spatial lines can also be used for cylindricity.

First, normal estimation [40] is performed for the cylindrical surface’s point cloud. The obtained unit normal is regarded as a new set of point clouds. Subsequently, the normal estimation is continued on the normal point cloud to obtain the initial axis direction, n(a,b,c), of the cylinder. As with straightness, by aligning the initial axis to the *Z*-axis via Equations (3)–(5), the control variables can be reduced from six to four, and the search area can be equally distributed in each direction. The process is also demonstrated in Figure 1 and Figure 2b.

4. Flatness

The distance function of the plane is $f(P_{i},a,b,c,d)$, where *d* is related to the plane position. Since the deviation zone is a relative distance, the parameter *d* has no effect, and the process is a three-dimensional optimization problem. We estimate the plane normal, n(a,b,c), using the least-squares method, and then the algorithm’s search area is determined by applying the Rodrigues rotation matrix, as shown in Figure 1 and Figure 2c.

### 2.2. Overview of HHO

**Figure 3:**
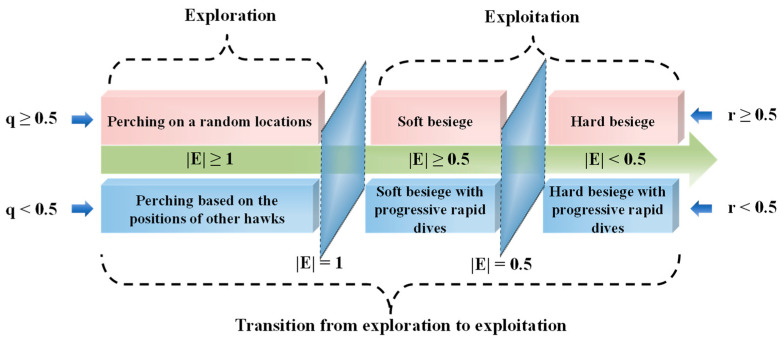
Six phases of HHO.

HHO is a population-based optimization algorithm that mimics the cooperative behavior of Harris hawks chasing prey (in most cases, rabbits) in nature. In the absence of prey, the hawks will randomly change position until the prey is found. When a rabbit is detected, the hawks will choose different strategies for besiegement, depending on the dynamic nature of the environment and the prey’s escape pattern. A switching tactic involves the best hawk (the leader) swooping at the prey and disappearing and the chase being continued by one of the party members. By means of this tactic, the detected rabbit is chased to exhaustion, resulting in a successful hunt. A total of six stages of HHO are plotted in Figure 3, and the specific HHO steps and mathematical model are described in the following subsection.

#### 2.2.1. Exploration Phase

In this phase, Harris hawks will choose two strategies to move with equal probability: one is to move based on the positions of other family members; the other is to perch on random tall trees. The process is modeled as follows:

(7) $$X\left(t+1\right)=\left\{ \begin{array}{l} X_{rand}(t)-r_{1}\left|X_{rand}(t)-2r_{2}X(t)\right|\;\;\;\;\;q\geq 0.5\\ \left(X_{prey}(t)-X_{ave}(t)\right)-r_{3}\left(r_{4}\left(UB-LB\right)+LB\right)\;q<0.5\; \end{array}\right.$$

where X(t+1) and X(t) represent the position vectors of the search agent in the *t* + 1 and *t* iterations, respectively, and each dimension represents a control variable; $q,r_{1},r_{2},r_{3},r_{4}$ are random variables inside (0, 1), which are updated in each iteration; $X_{rand}(t)$ is the position vector of a random individual; $X_{prey}(t)$ denotes the rabbit’s position vector, which is the best agent; *LB* and *UB* are the lower and upper bounds of the control variables; and $X_{ave}(t)$ is the average position vector of the current search agents, which is calculated using the following equation:

(8) $$X_{ave}(t)=\frac{1}{N}\sum_{i=1}^{N}X_{i}(t)$$

where *N* denotes the total number of hawks and $X_{i}(t)$ indicates the location of each hawk in iteration *t*.

#### 2.2.2. Transition from Exploration to Exploitation

The prey’s energy decreases over time during escape. HHO can transition from exploration to exploitation and choose different exploitation strategies based on the prey’s escape energy. The prey’s escape energy is modeled as a time-varying stochastic parameter as follows:

(9) $$E=2E_{0}\left(1-\frac{t}{T}\right)$$

where *E* indicates the remaining energy of the rabbit in the escape process, *T* denotes the maximum number of iterations, and $E_{0}$ is the initial state of its energy generated randomly inside the interval (−1, 1) in each iteration. Thus, if |E|≥1, this means that the rabbit has enough energy to escape, so the HHO will perform diverse exploration operations, and if |E|<1, the rabbit is weak, so the algorithm will try to exploit the neighborhood of the solutions.

#### 2.2.3. Exploitation Phase

For this phase, according to the escape behaviors of the prey and the chasing strategies of Harris hawks, four possible strategies are proposed for HHO to model the attacking stage. Let r be the chance that the prey escapes successfully (r<0.5) or escapes unsuccessfully (r≥0.5).

1. Soft Besiegement

When r≥0.5 and E≥0.5, the prey still has enough energy to escape dangerous situations. At this time, the hawks will perform a soft besiegement to continuously exhaust the rabbit’s energy and prevent it from making random misleading jumps by encircling it softly. If the jump strength of a rabbit is denoted as $J=2\left(1-r_{5}\right)$, where $r_{5}$ is a random number inside (0, 1), this behavior can be modeled according to the following rules:

(10) $$X(t+1)=\Delta X(t)-E\left|JX_{prey}(t)-X(t)\right|$$

(11) $$\Delta X(t)=X_{prey}(t)-X(t)$$

where ΔX(t) is the difference between the prey’s position vector and the current location in iteration *t*.

2. Hard Besiegement

When r≥0.5 and E<0.5, the intended prey exhausts the energy, and the hawks finally perform the surprise pounce. In this situation, the current position is updated using Equation (12):

(12) $$X(t+1)=X_{prey}-E\left|\Delta X(t)\right|$$

3. Soft Besiegement with Progressive Rapid Dives

When r<0.5, the prey has a chance to escape successfully, and the hawks will adopt the chasing strategies of soft besiegement and hard besiegement, but their doing so is more intelligent than in the previous case. By utilizing the concept of Levy flight (LF), the real zigzag deceptive motions of prey are mimicked and the hawks will progressively adjust their location and directions through rapid dives. When the prey has sufficient energy (E≥0.5), the process is expressed as follows:

(13) $$X(t+1)=\left\{ \begin{array}{l} Y\;if\;f(Y)<f(X(t))\\ Z\;if\;f(Z)<f(X(t))\; \end{array}\right.$$

(14) $$Y=X_{prey}(t)-E\left|JX_{prey}(t)-X(t)\right|$$

(15) Z=Y+S×LF(D)

where *D* is the dimension of the problem, *S* is a random vector of size 1×D, and *LF* is the Levy flight function, which is modeled as follows:

(16) $$LF(D)=0.01\times \frac{u\times \sigma }{{\left|v\right|}^{\frac{1}{\beta }}},\sigma ={\left(\frac{\Gamma (1+\beta )\times sin\left(\frac{\pi \beta }{2}\right)}{\Gamma \left(\frac{1+\beta }{2}\right)\times \beta \times 2^{\left(\frac{\beta -1}{2}\right)}}\right)}^{\frac{1}{\beta }},\beta =1.5$$

where *u* and *v* are random numbers in the range (0, 1).

4. Hard Besiegement with Progressive Rapid Dives

Similarly, when E<0.5, the prey does not have enough energy to escape, and the rapid dive strategy with LF is modeled as follows:

(17) $$X(t+1)=\left\{ \begin{array}{l} Y\;if\;f(Y)<f(X(t))\\ Z\;if\;f(Z)<f(X(t))\; \end{array}\right.$$

(18) $$Y=X_{prey}(t)-E\left|JX_{prey}(t)-X_{ave}(t)\right|$$

(19) Z=Y+S×LF(D)

### 2.3. Overview of SSA

In the foraging behavior of the salp swarm, the group is divided into two parts, the leader and the followers. The first individual is considered the leader, and the other individuals form the main body of the chain and are called the followers. Newtonian mechanical analysis is used to model the leader’s and the followers’ movements separately. The leader position vector is updated using the following equation:

(20) $$X_{1,j}=\left\{ \begin{array}{l} F_{j}+c_{1}\times \left(c_{2}\times \left(UB_{j}-LB_{j}\right)+LB_{j}\right),c_{3}\geq 0.5\\ F_{j}-c_{1}\times \left(c_{2}\times \left(UB_{j}-LB_{j}\right)+LB_{j}\right),c_{3}<0.5 \end{array}\right.$$

where dimension j={1,2,…,D}, $F={\left[F_{1},F_{2},\ldots F_{D}\right]}^{T}$ denotes the position vector of the target agent or the current best solution, $c_{2}$ and $c_{3}$ are the adaptive tuning parameters between (0, 1), and $X_{1,j}$ are the position vectors of the leader in the *j*th dimension. $c_{1}$ is an important factor in controlling exploration and exploitation and is calculated by means of the following equation:

(21) $$c_{1}=2e^{-{\left(\frac{4t}{T}\right)}^{2}}$$

The follower’s update formula is expressed as:

(22) $$X_{i,j}=\frac{X_{i,j}+X_{i-1,j}}{2},i=2,3,\ldots ,N$$

As the leader moves, the fluctuation of the leader’s position change is transmitted to each follower step by step with the salp chain. The leader continuously explores the space around the moving food source, F. This significantly enhances the exploration ability of SSA and enables the salp chain to catch up with the moving food source and finally complete the foraging behavior.

## 3. The Proposed Optimizer

To solve the problem of HHO easily falling into a local optimum and having slow convergence, this paper proposes an improved HHO optimization algorithm. The IHHO focuses on two areas of improvement: exploration strategy selection and exploration capabilities. The following is a specific description of the improved HHO algorithm, and the pseudocode is given in Algorithm 1.

### 3.1. Exploration Based on Average Fitness

In optimization techniques, random operators are often used to determine the update strategy of the search agent due to their random nature. However, in HHO, strategy selection based on random operators may conflict with the actual situation. In detail, when the hawk is very near to the prey $\left(f(X_{i})>f(X_{prey})\right)$, it incorrectly moves to a random location due to a better perching chance (q≥ 0.5), but the correct strategy is to perch with other family members. When the hawk is far from the prey $\left(f(X_{i})\gg f(X_{prey})\right)$, it incorrectly perches with other family members due to a lower perching chance (q<0.5), but the correct strategy is to move to a random high tree to perch suddenly. Therefore, we replace the random operator, *q*, of HHO in the exploration phase with the average fitness to solve the conflict problem when choosing between two strategies. Let us define the average fitness, $f_{ave}$, of the search agents’ locations as:

(23) $$f_{ave}=\frac{1}{N}\sum_{i=1}^{N}f(X_{i})$$

Then, Equation (7) becomes the following equation:

(24) $$X\left(t+1\right)=\left\{ \begin{array}{l} X_{rand}(t)-r_{1}\left|X_{rand}(t)-2r_{2}X(t)\right|f(X_{i})<f_{ave}\\ \left(X_{prey}(t)-X_{ave}(t)\right)-r_{3}\left(r_{4}\left(UB-LB\right)+LB\right)f(X_{i})\geq f_{ave} \end{array}\right.$$

where $f(X_{i})$ denotes the fitness value of the individual agent and *N* is the number of agents.
**Algorithm 1**: Pseudocode of IHHO→ Set the initial iteration *t* = 1→ Initialize the random search space $X_{i}=(x_{i}^{1},x_{i}^{2},\ldots x_{i}^{D})$ for the *i*th hawk of the *D* dimension problem within the search boundary [*UB*, *LB*]While t≤T
  A. Evaluate the fitness value, $f(X_{i})$, of all N hawks  B. Find the best agent to be the leader and use the rest as followers  C. Update the leader and followers using Equation (20) and Equation (26)  D. If the SSA individual is better, replace the corresponding HHO individual and update the fitness value  E. Calculate the average fitness, $f_{ave}$, using Equation (23)  F. Label the best prey location as $X_{prey}$  G. For *i* = 1, 2, … *N* (each hawk)   a. Update the escaping energy *E* by Equation (9)   b. If (|E|≥1)       → Randomly choose one hawk location as $X_{rand}$ from the search space       → Calculate the mean position vector $X_{ave}$ using Equation (8)       → Update the new location using Equation (24)         Elseif (|E|<1)
       → Generate a random escaping chance of prey *r* in the range [0, 1]         If (r≥0.5 and |E|≥0.5)
         Update the new location using Equation (10)       Elseif (r≥0.5 and |E|<0.5)
         Update the new location using Equation (12)       Elseif (r<0.5 and |E|≥0.5)
         Update the new location using Equation (13)       Elseif (r<0.5 and |E|<0.5)
         Update the new location using Equation (17)       End (If)      End (If)    End (For)  H. Amend the search space $X_{i}$ for *i* = 1, 2, … *N* based on the search boundary *UB* and *LB*  *I. t = t+1*End (While)→ Return the best location $X_{prey}$


### 3.2. Nonlinear Inertia Weight

Note that the values of the inertia weights affect the algorithm’s efficiency. Larger inertia weights enhance the global search capability of the algorithm, and, conversely, smaller inertia weights enhance the local search capability. To solve the problem of low convergence accuracy and slow convergence of the traditional SSA algorithm, a nonlinear inertia weight is introduced in the update formula of the follower salp to evaluate the degree of interindividual influence, with values nonlinearly transformed between 0.9 and 0.4. The proposed nonlinear inertia weights are as follows:

(25) w=(winit−wend−k)e11+tutmax

where $w_{init}$ is the initial inertia weight, $w_{end}$ is the inertia weight for the maximum number of iterations, and *k* and *u* are control coefficients that regulate the range of *w*. After sufficient experiments, taking $w_{init}=0.98$, $w_{end}=0.4$, *k* = 0.21, and *u* = 11.2. the new follower update rule is:

(26) $$X_{i,j}=wX_{i,j}+X_{(i-1),j},i=2,3,\ldots ,N$$

The addition of a nonlinear inertia weight enhances the global search ability in the early stage compared to the previous averaging strategy with a fixed weight of 0.5. It enhances the local search ability of the algorithm in the later stage and balances the exploration and exploitation of the SSA.

### 3.3. Hybrid SSA

Combining algorithms has become a trend in optimization research in recent years. The superior explorative ability of SSA can fill the gap in the exploration of conventional HHO. Therefore, this paper embeds the SSA with a nonlinear inertia weight into HHO to improve the diversity of hawks while retaining its inherent excellent convergence and exploitation capabilities.

Specifically, before updating the search agent through the HHO mechanism, the space around the current best agent is explored with SSA to determine whether a better agent exists, and, if so, the position of the HHO individual is updated to the SSA individual. Otherwise, it remains unchanged. Subsequently, the individual with the smallest objective function value is selected as the prey’s position vector, $X_{prey}$, in $X_{HHO}$, $X_{SSA}$.

(27) $$X_{prey}=\min\{f\left(X_{HHO}\right),f\left(X_{SSA}\right)\}$$

## 4. Performance Evaluation of the IHHO Algorithm

### 4.1. Benchmark Functions and Compared Algorithms

In this section, the proposed improved HHO algorithm (IHHO) is investigated using a set of 23 diverse classical benchmark functions from [37]. The benchmark functions can be divided into three categories: unimodal (of which there are seven), multimodal with varied dimensions (of which there are six), and multimodal with fixed dimensions (of which there are ten). The unimodal sets have a globally unique solution suitable for revealing the optimizer’s exploitation capabilities, whereas the multimodal sets have multiple optima that disclose the explorative capability and local optimum avoidance potentials of the proposed optimizer. The mathematical descriptions of the benchmark functions are shown in Table 1, Table 2 and Table 3.

To investigate the performance of IHHO, in addition to comparison with traditional HHO, other well-recognized swarm intelligence methods, such as PSO [41], GA [42], DE [43], TLBO [44], ABC [45], CS [46], WOA [47], and SSA [38], were also compared. The quantitative analysis included the average value and standard deviation (std. dev.), and the qualitative analysis included the prey position, search history, trajectory, diversity history, average fitness history, boxplot and convergence curves. Furthermore, the nonparametric statistical results of the Wilcoxon signed-rank test and Friedman test were introduced to detect the substantial differences between optimizers. The significance level was set at 0.05. The Wilcoxon signed-rank test categorized the IHHO calculations as significantly better (+), equal (=), or significantly worse (−) by *p*-values. Further statistical comparisons were made by applying the Friedman test for average ranking performance (expressed as ARV).

### 4.2. Experimental Setup

In this study, the following control variables were adopted: the maximum number of iterations equaled 500 iterations, and the number of search agents equaled 30. The dimension of the function was set to 30 if it was a non-fixed problem. The parameter settings used for various optimization algorithms are reported in Table 4. Every method applied 30 independent runs to avoid the effect of randomness in MATLAB2016a using a Windows 10 64-bit Intel (R) Core (TM) i7-11800 h@2.30 GHz with 16 GB.

**Figure 4:**
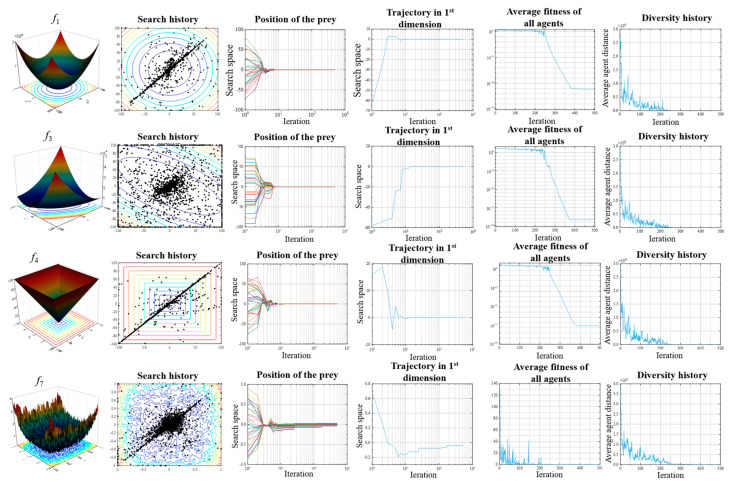
Qualitative results of unimodal classical benchmark functions *f*_1_, *f*_3_, *f*_4_, and *f*_7_.

The trajectory curve represents the change of the first agent in the first dimension during 500 iterations. It can be observed from Figure 4 that the curve reached the optimal solution after oscillations in the initial iteration, which reveals the exploration behavior of the algorithm. For complex functions, the fluctuation will be correspondingly more significant.

**Figure 5:**
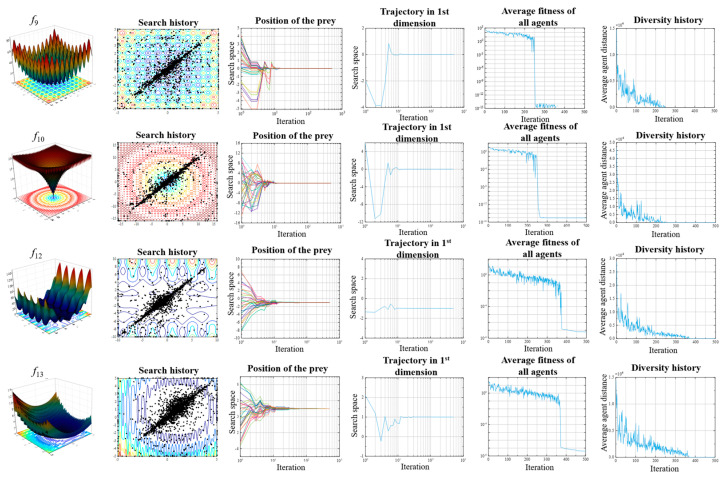
Qualitative results of multimodal classical benchmark functions *f*_9_, *f*_10_, *f*_12_, and *f*_13_.

The average fitness is a measure of the collaborative behavior of the hawk. The average fitness in Figure 4 and Figure 5 decreases with iterations, which indicates that all hawks update to a better position with an increasing number of generations. 

The diversity history reveals the transition between the exploration and exploitation of search agents. In this paper, diversity is calculated by the Euclidean distance between *N* hawks. If the *i*th agent of the *D*-dimensional problem is represented as $X_{i}=(x_{i,1},x_{i,2},\ldots ,x_{i,D})$, then at a specific iteration t, the diversity is calculated as:

(28) $$diversity=\sum_{i=1}^{N}\sum_{j=1}^{N}\left(\sum_{k=1}^{D}\sqrt{{\left(x_{i,k}(t)-x_{j,k}(t)\right)}^{2}}\right)$$

As can be seen from the diversity history diagrams in Figure 4 and Figure 5, there is more diversity in the initial stage than in the later stage of the optimization algorithm. The IHHO algorithm performs more exploration in the initial stage, while in the later stages it performs more exploitation. Moreover, the curve tends to zero as the iterations proceed, revealing that the proposed algorithm strikes a good balance between exploration and exploitation.

### 4.3. Comparison with Conventional Swarm-Based Algorithms

**Figure 6:**
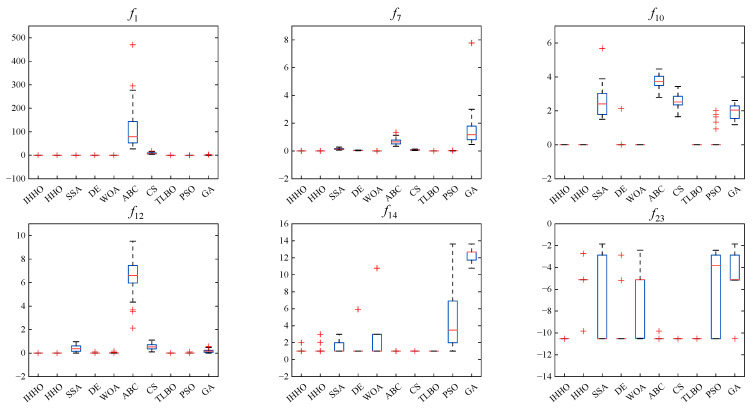
Boxplots of the six classical benchmark functions *f*_1_, *f*_7_, *f*_10_, *f*_12_, *f*_14_, and *f*_23_.

**Figure 7:**
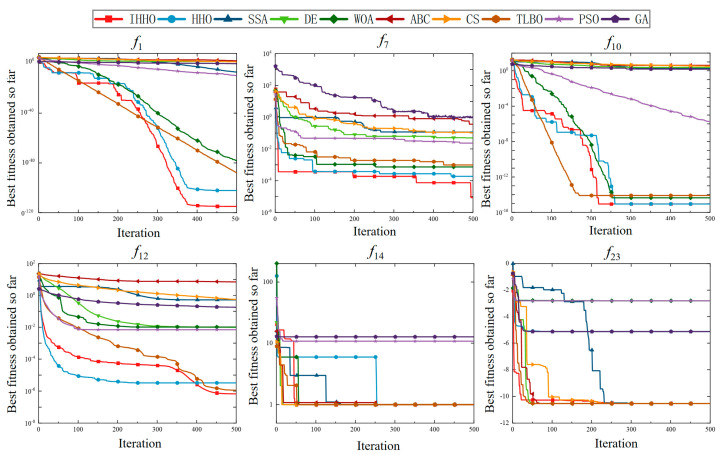
The convergence curves of the six classical benchmark functions.

In this section, the statistical results of the conventional swarm-based algorithm and the proposed IHHO for 23 benchmark problems are presented in Table 5. The results expose the statistical outcomes in terms of average values and standard deviations. The number of dimensions for all problems was 30, except for the fixed-dimensional multimodal problems *f*_14_–*f*_23_. The best values are in bold in Table 5, while their statistical significance can be observed in Table 6. In addition, Figure 6 and Figure 7 demonstrate the boxplots and convergence curves for unimodal functions (*f*_1_, *f*_4_), multimodal benchmark functions with varied dimensions (*f*_8_, *f*_9_), and multimodal benchmark functions with fixed dimensions (*f*_15_, *f*_21_) in repeated experiments.

From the statistical results listed in Table 5, the proposed IHHO algorithm had a significant advantage over the other algorithms in terms of average values and standard deviations. For most functions, IHHO was able to find the best solutions, even the optimal ones, except for *f*_6_, *f*_8_, and *f*_20_. For *f*_6_, the performance of IHHO was worse than that of PSO and SSA but far better than that of traditional HHO algorithms. For *f*_8_, the results were better than those of the other nine algorithms and slightly inferior to those of the HHO algorithm, while, for *f*_20_, IHHO also generated the best solution. From the perspective of standard deviations, the standard deviations of IHHO were the lowest for 15 functions, even though the standard deviations for *f*_9_, *f*_10_, and *f*_11_ were 0. Although the standard deviations of IHHO were poorer than those of DE, CS, and TLBO for *f*_16_–*f*_19_ when the same average values were obtained, it outperformed the other algorithms. Meanwhile, IHHO came next in performance to the first method for *f*_14_. Therefore, IHHO can achieve satisfactory solutions with guaranteed accuracy and stability.

According to the *p*-values of the Wilcoxon rank-sum tests for analyzing the significant differences of the paired algorithms in Table 6, the performance of IHHO had significant positive differences compared to the other algorithms with respect to the test results for the 23 benchmark functions, except for DE, CS, and TLBO. Although the statistical results for IHHO were significantly worse than those for DE, CS, and TLBO for *f*_16_–*f*_19_, the main reason was the difference in the standard deviations. As analyzed before, IHHO was superior to the rest of the methods with respect to standard deviation, so it can still be considered that IHHO can obtain high-quality solutions. Additionally, from the overall significant statistical results of the Wilcoxon rank-sum tests for all functions, the worst case IHHO produced 17 significantly better, 1 equal, and 5 significantly worse results (TLBO), and in the best case IHHO overwhelmingly succeeded for all benchmark functions (GA). From the statistical results of the Friedman test, the best ARV of 1.61 was obtained for IHHO, which is also consistent with the Wilcoxon rank-sum test results; IHHO was far superior to TLBO in second place with a ranked value of 4.04. Therefore, it can be concluded that the IHHO algorithm is an improvement on the HHO algorithm with considerable advantages over the other nine competitive swarm-based algorithms.

The boxplot diagrams of the classical test functions are shown in Figure 6. As can be seen from the boxplots, IHHO consistently outperformed or equaled the other optimization algorithms, while HHO underperformed for the non-scalable function *f*_23_. In addition, the TLBO algorithm also showed strong consistency. On average, IHHO showed results comparable to those of other optimization algorithms using the boxplot representation.

The convergence curves for six classical benchmark functions are presented in Figure 7. Based on the observation, IHHO ranked first for *f*_1_, *f*_7_, *f*_10_, and *f*_12_ and performed the same as TLBO for *f*_23_. For the test function *f*_14_, IHHO ranked second with TLBO and WOA—worse than CS, DE, and ABC, but better than the other algorithms. Regarding the overall performance of IHHO for 23 benchmark problems, which are combinations of unimodal and multimodal problems designed to test exploration and exploitation capabilities, it can be stated that IHHO can be used for function optimization.

### 4.4. Discussion

In this section, the effectiveness of the improved HHO algorithm is verified by 23 benchmark functions. It is important to note that all the experiments were executed under the same conditions. First, a qualitative analysis of the IHHO algorithm was performed. By analyzing the eight benchmark functions in five aspects, namely, search history, prey position, trajectory, average fitness value, and diversity, it was demonstrated that the IHHO algorithm can balance exploration and exploitation, thus avoiding falling into local solutions and finding the optimal value. Hence, IHHO can perform the optimization search for complex nonlinear optimization problems. Second, to comprehensively assess the advantages of the proposed algorithm, the IHHO algorithm was compared with several swarm-based methods in six aspects: average values, standard deviations, Wilcoxon rank-sum tests, Friedman tests, boxplot diagrams, and convergence curves. The comparative outcomes of all cases revealed that the developed IHHO optimizer, which fuses the average fitness exploration strategy and the nonlinear inertia weight SSA algorithm, obtained better overall performance and converged faster than the alternatives.

Accordingly, the proposed optimizer significantly enhances the optimization capability compared to several other classical optimization algorithms. This is because the SSA mechanism makes the search agents better diversified while taking full advantage of the excellent intensification capability of the original HHO algorithms. Although other variants of HHO embedded within the SSA mechanism are available, the exploration strategy based on the average fitness value and the introduction of nonlinear inertia weights described in this paper is innovative and further enhances the coordination of intensification and diversification, with excellent results. However, similar to other swarm-based optimizers, there are also some limitations to the proposed optimizer. First of all, it may expend more time on optimization because of the addition of the SSA exploration mechanism. Second, the range of nonlinear inertia weights may need to be adjusted in some cases. Therefore, there is a need to harmonize efficiency and accuracy when solving problems with real-time requirements.

## 5. The Application of IHHO in Form Deviation-Zone Evaluation

Traditional form deviation-zone evaluation suffers from the problems of difficulty in generating solutions, poor generality, and lack of solution accuracy, while other metaheuristic intelligent optimization algorithms have a wide variety of algorithms, each containing many variants, and each having its own advantages and disadvantages. Therefore, the goal of this study was to find an algorithm with strong global optimization capability, less parameter adjustment, and high accuracy for error evaluation.

It has been shown that the HHO algorithm has fewer parameters, is simple in principle, is more exploratory and adaptable in global optimization, and outperforms many well-known intelligent optimization methods, such as PSO, GWO, CS, DE, and WOA. Therefore, its application to form deviation-zone evaluation satisfies the requirement of fewer parameter adjustments and has some advantages over other methods in terms of optimization capability and optimization accuracy. Although there are still problems of early convergence, poor optimization accuracy, and weak global search capability, they have been improved by various measures.

### 5.1. Comparison of Data in the Literature

**Figure 8:**
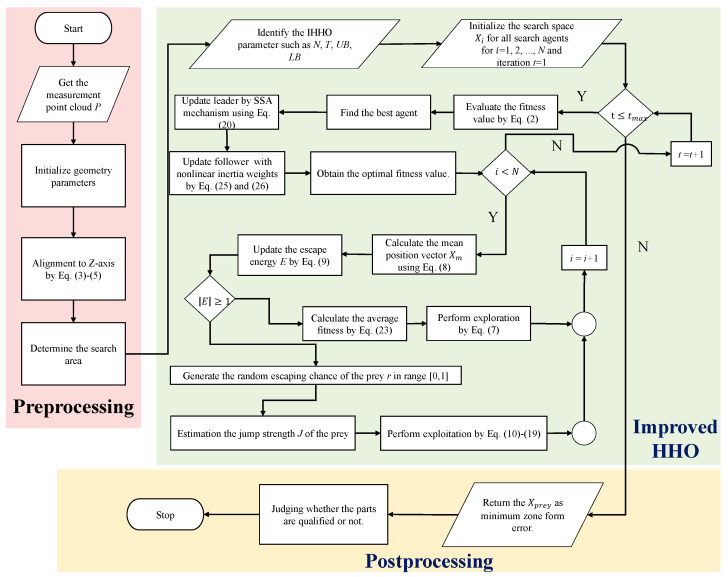
Flowchart of IHHO-based deviation-zone evaluation.

**Figure 9:**
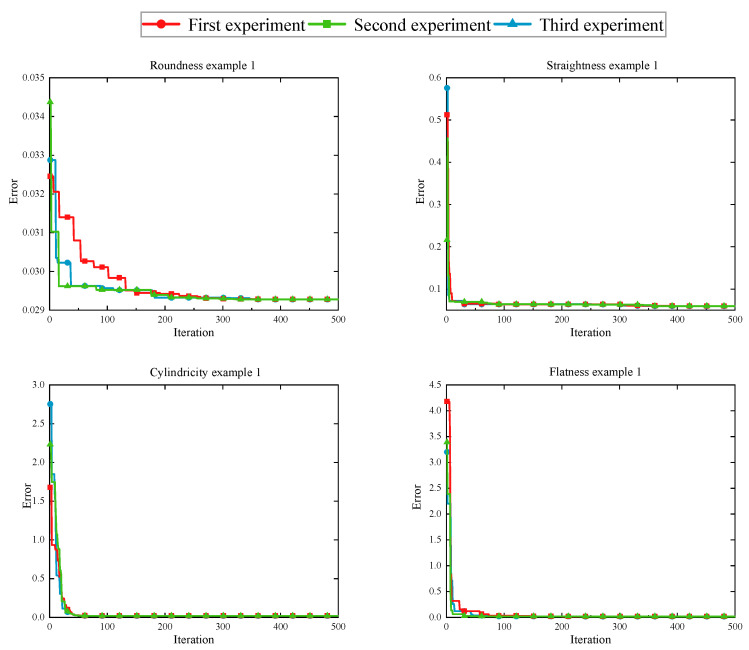
The convergence curves relative to the number of iterations.

To evaluate the availability of IHHO in form deviation-zone evaluation, we benchmarked the proposed IHHO by reference to data in the literature. The flowchart of IHHO applied to solve the deviation-zone evaluation problem is shown in Figure 8. The population size was set to 30, the maximum number of iterations was 500, and the optimization dimension and search area for the corresponding problem were shown in Section 2.1. The algorithm was run 30 times independently using MATLAB2016a software, and the average result was taken as the corresponding form error. Table 7 shows the evaluation results of the algorithms reported in the literature and those obtained by IHHO. The results list the number of points, reported minimum-zone errors, IHHO evaluation minimum-zone errors, least-squares evaluation errors, and relative differences. In addition, the convergence curves of three randomly selected experiments are shown in Figure 9 to visualize the working process of the IHHO algorithm for deviation-zone evaluation.

From Table 7, the average evaluation results for the IHHO algorithm in the four types of deviation zones are more accurate or equal to the reported MZs in the literature, except for example 2 of flatness, and significantly improved compared to the least-squares method. In particular, the straightness evaluation error of example 2 improved by 25.65% compared to the reported results. As can be seen from the convergence diagram in Figure 9, the optimal solution was found with only 50 iterations on the dataset, except for the roundness error evaluation, which reached convergence in approximately 150 iterations. The trends were essentially the same for the three randomly selected experiments. Therefore, it can be tentatively concluded that the proposed IHHO optimization algorithm works well in deviation-zone evaluation and can meet the needs of high-precision evaluation in engineering.

### 5.2. Engineering Applications

**Figure 10:**
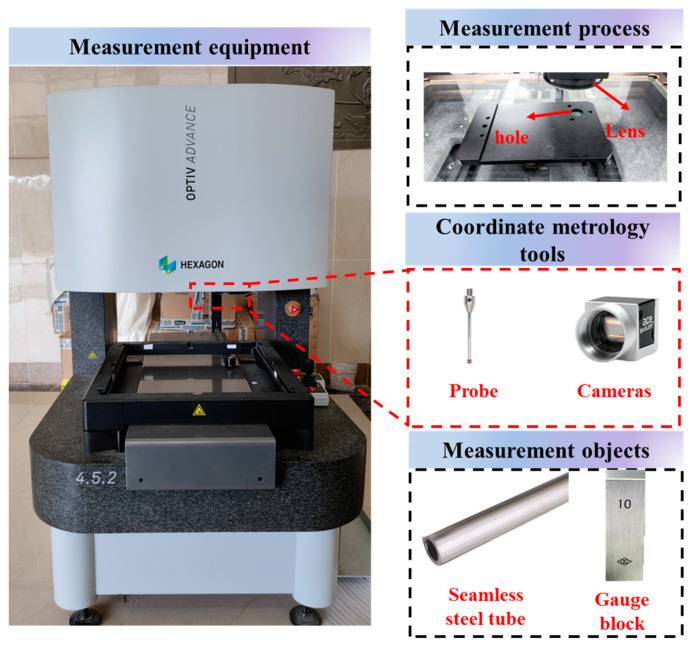
(Data acquisition equipment and measurement objects).

To further validate the advantages of the IHHO algorithm applied to deviation-zone evaluation, the surface of a seamless steel tube was measured by a hexagon image-probe hybrid measuring device, MSOC-03-2C. With the probe system, eight sets of section data and corresponding center coordinates were collected to assess the cylindricity and axis straightness of the seamless steel tube. With the vision system, the coordinates of the cross-section of the steel tube hole were collected, filtered, and downsampled for roundness evaluation. Furthermore, the measuring surface of a 10 mm gauge block was collected with a probe for flatness evaluation. The experimental equipment and objects are shown in Figure 10.

**Figure 11:**
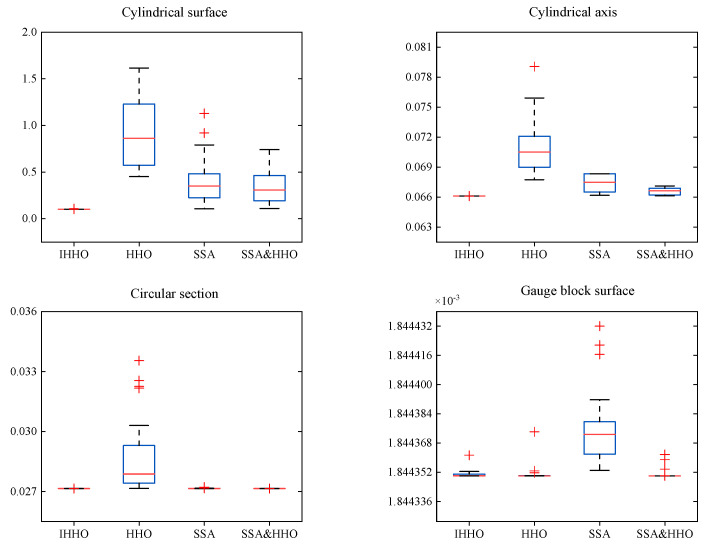
Boxplots of the evaluation results for the seamless steel pipe and gauge block data of different algorithms.

**Figure 12:**
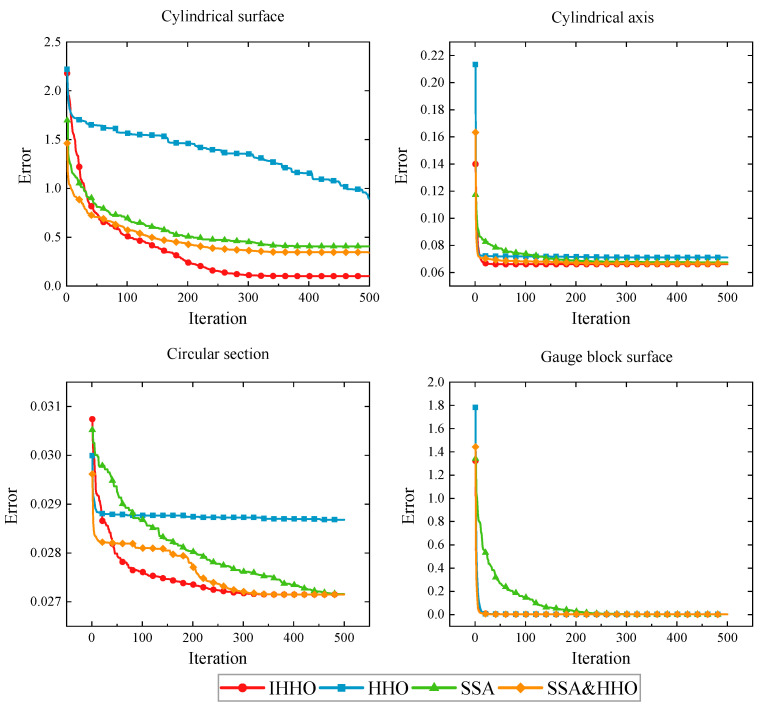
The average convergence curves for the seamless steel tube and gauge block data.

**Figure 13:**
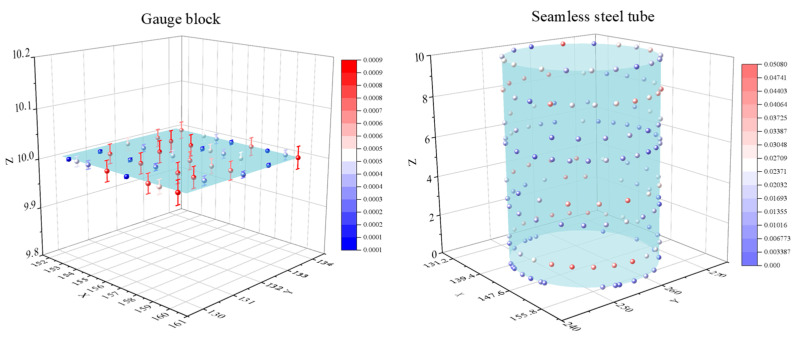
The error maps for the seamless steel tube and gauge block surface.

Based on the deviation-zone model developed in Section 2.1, the above acquisition data were evaluated using the IHHO, HHO, SSA, SSA&HHO [34], and least-squares methods. To thoroughly verify the convergence property of the algorithm, the maximum number of iterations *T* = 500 and *N* = 30. The experiments were repeated 30 times for each data group to remove accidental errors. The results are summarized in Table 8. The boxplots and average convergence curves for the different algorithms relative to the number of iterations are plotted in Figure 11 and Figure 12. The error maps of the gauge block surface and the seamless tube surface are shown in Figure 13.

As shown in Table 8, in the cylindricity evaluation, the error was 0.1013 mm, which is much higher than that of the other algorithms. For straightness, the SSA&HHO and IHHO algorithms were more effective, while SSA and HHO were poor. HHO performed the worst in the roundness evaluation, while the rest of the algorithms performed similarly. In the flatness evaluation, all algorithms achieved the same results. According to the boxplot diagram in Figure 11, the IHHO fluctuations were lower than those of the other methods in 30 independent runs, while the rest of the algorithms showed performance differences when evaluating different form errors.

## 6. Conclusions

This paper proposes an improved Harris hawks optimization algorithm based on the average fitness exploration strategy and nonlinear inertia weights (SSA). The idea of using average fitness in the exploration phase provided us with a solution to the strategy selection conflict caused by randomness. Furthermore, the introduction of nonlinear inertia weights further enhanced the global search capability of the SSA algorithm, enabling it to give full play to its advantages when embedded in HHO and compensating for the shortcomings of the HHO exploration phase. Although the computational complexity of the IHHO algorithm was slightly higher than that of the HHO algorithm, the convergence of the IHHO algorithm was faster than HHO in terms of the number of iterations and function evaluation results. The IHHO algorithm was thoroughly compared with the well-established optimization algorithms, and the results showed that the IHHO algorithm outperformed the other optimization algorithms. With respect to the engineering problem, the IHHO algorithm was compared with other algorithms using data reported in the literature and collected data to verify its effectiveness and superiority in determining form errors. The results show that IHHO is applicable to the deviation-zone evaluation problem and can give accurate and reliable form error evaluation results. However, this paper does not deal with free surfaces without a specific functional expression. Therefore, in future studies, a promising direction would be to evaluate the deviation zone based on the CAD model and the collected discrete points.

## Figures and Tables

**Table 1:** Unimodal benchmark functions.

Name	Function	Range	$f_{\min}$
Sphere Function	$f_{1}(x)=\sum_{i=1}^{n}x_{i}^{2}$	[−100, 100]	0
Schwefel’s Problem 2.22	$f_{2}(x)=\sum_{i=1}^{n}\left|x_{i}\right|+\prod_{i=1}^{n}\left|x_{i}\right|$	[−10, 10]	0
Schwefel’s Problem 1.2	$f_{3}(x)={\sum_{i=1}^{n}\left(\sum_{j=1}^{i}x_{j}\right)}^{2}$	[−100, 100]	0
Schwefel’s Problem 2.21	$f_{4}(x)=\max_{i}\{\left|x_{i}\right|,1\leq i\leq n\}$	[−100, 100]	0
Generalized Rosenbrock’s Function	$f_{5}(x)=\sum_{i=1}^{n-1}\left[100{\left(x_{i+1}-x_{i}^{2}\right)}^{2}+{\left(x_{i}-1\right)}^{2}\right]$	[−30, 30]	0
Step Function	$f_{6}(x)=\sum_{i=1}^{n-1}{\left(\left|x_{i}+0.5\right|\right)}^{2}$	[−100, 100]	0
Quartic Function	$f_{7}(x)=\sum_{i=1}^{n-1}ix_{i}^{4}+\mathrm{random}[0,1]$	[−1.28, 1.28]	0

**Table 2:** Multimodal benchmark functions.

Name	Function	Range	$f_{\min}$
Generalized Schwefel’s Problem 2.26	$f_{8}(x)=\sum_{i=1}^{n}-x_{i}\sin\left(\sqrt{\left|x_{i}\right|}\right)$	[−500, 500]	−418.9829 × n
Generalized Rastrigin’s Function	$f_{9}(x)=\sum_{i=1}^{n}\left[x_{i}^{2}-10\cos\left(2\pi x_{i}\right)+10\right]$	[−5.12, 5.12]	0
Ackley’s Function	$f_{10}(x)=-20\exp\left(-0.2\sqrt{\frac{1}{n}\sum_{i=1}^{n}x_{i}^{2}}\right)-\exp\left(\frac{1}{n}\sum_{i=1}^{n}\cos\left(2\pi x_{i}\right)\right)+20+e$	[−32, 32]	0
Generalized Griewank’s Function	$f_{11}(x)=\frac{1}{4000}\sum_{i=1}^{n}x_{i}^{2}-\prod_{i=1}^{n}\cos\left(\frac{x_{i}}{\sqrt{i}}\right)+1$	[−600, 600]	0
Generalized Penalized Function 1	$\begin{array}{l} f_{12}(x)=\frac{\pi }{n}\left\{10\sin\left(\pi y_{1}\right)+\sum_{i=1}^{n}{\left(y_{i}-1\right)}^{2}\left[1+10\sin^{2}\left(\pi y_{i+1}\right)\right]+{\left(y_{n}-1\right)}^{2}\right\}+\sum_{i=1}^{n}u\left(x_{i},10,100,4\right)\\ y_{i}=1+\frac{x_{i}+1}{4},u\left(x_{i},a,k,m\right)=\left\{ \begin{array}{l} k{\left(x_{i}-a\right)}^{m}\;\;x_{i}>a\\ 0\;\;\;\;-a<x_{i}<a\\ k{\left(-x_{i}-a\right)}^{m}\;\;x_{i}<-a \end{array}\right. \end{array}$	[−50, 50]	0
Generalized Penalized Function 2	$\begin{array}{l} f_{13}(x)=0.1\left\{\sin^{2}\left(3\pi x_{1}\right)+\sum_{i=1}^{n}{\left(x_{i}-1\right)}^{2}\left[1+\sin^{2}\left(3\pi x_{1}+1\right)\right]+{\left(x_{n}-1\right)}^{2}\left[1+\sin^{2}\left(2\pi x_{n}\right)\right]\right\}\\ +\sum_{i=1}^{n}u(x_{i},5,100,4) \end{array}$	[−50, 50]	0

**Table 3:** Fixed-dimension multimodal benchmark functions.

Name	Function	Dimension	Range	$f_{\min}$
Shekel’s Foxholes Function	$f_{14}(x)={\left(\frac{1}{500}+\sum_{j=1}^{25}\frac{1}{j+\sum_{i=1}^{2}{\left(x_{i}-a_{ij}\right)}^{6}}\right)}^{-1}$	2	[−65.536, 65.536]	1
Kowalik’s Function	$f_{15}(x)=\sum_{i=1}^{11}{\left[a_{i}-\frac{x_{i}\left(b_{i}^{2}-b_{i}x_{2}\right)}{b_{i}^{2}+b_{i}x_{3}+x_{4}}\right]}^{2}$	4	[−5, 5]	0.0003075
Six-Hump Camel-Back Function	$f_{16}(x)=4x_{1}^{2}-2.1x_{1}^{2}+\frac{1}{3}x_{1}^{6}+x_{1}x_{2}-4_{2}^{2}+4x_{2}^{4}$	2	[−5, 5]	−1.0316285
Branin Function	$f_{17}(x)={\left(x_{2}-\frac{5.1}{4\pi^{2}}x_{1}^{2}+\frac{5}{\pi }x_{1}-6\right)}^{2}+10\left(1-\frac{1}{8\pi }\right)\cos x_{1}+10$	2	[−5, 10] × [0, 15]	0.398
Goldstein–Price Function	$\begin{array}{l} f_{18}(x)=\left[1+{\left(x_{1}+x_{2}+1\right)}^{2}\left(19-14x_{1}+3x_{1}^{2}-14x_{2}+6x_{1}x_{2}+3x_{2}^{2}\right)\right]\times \\ \left[30+{\left(2x_{1}-3x_{2}\right)}^{2}\left(18-32x_{1}+12x_{1}^{2}+48x_{2}-36x_{1}x_{2}+27x_{2}^{2}\right)\right] \end{array}$	2	[−2, 2]	3
Hartman’s Family Function 1 (*N* = 3)	$f_{19}(x)=-\sum_{i=1}^{4}c_{i}\exp\left(-\sum_{j=1}^{3}a_{ij}{\left(x_{j}-p_{ij}\right)}^{2}\right)$	3	[0, 1]	−3.86
Hartman’s Family Function 2 (*N* = 6)	$f_{20}(x)=-\sum_{i=1}^{4}c_{i}\exp\left(-\sum_{j=1}^{6}a_{ij}{\left(x_{j}-p_{ij}\right)}^{2}\right)$	6	[0, 1]	−3.32
Shekel’s Family Function 1 (*N* = 5)	$f_{21}(x)=-\sum_{i=1}^{5}{\left[\left(X-a_{i}\right){\left(X-a_{i}\right)}^{T}+c_{i}\right]}^{-1}$	4	[0, 10]	−10.1532
Shekel’s Family Function 2 (*N* = 7)	$f_{22}(x)=-\sum_{i=1}^{7}{\left[\left(X-a_{i}\right){\left(X-a_{i}\right)}^{T}+c_{i}\right]}^{-1}$	4	[0, 10]	−10.4028
Shekel’s Family Function 3 (*N* = 10)	$f_{23}(x)=-\sum_{i=1}^{10}{\left[\left(X-a_{i}\right){\left(X-a_{i}\right)}^{T}+c_{i}\right]}^{-1}$	4	[0, 10]	−10.5363

**Table 4:** Parameter setting of various optimization algorithms.

Algorithm	Parameters
DE [43]	Scaling factor, *F* = 0.5 and crossover probability, *C_r_* = 0.9
WOA [47]	*a* = [0, 2], *b* = 1, and *l* = [−1, 1]
ABC [45]	Abandonment limit = 0.6 × *D* × *N*
CS [46]	Abandon probability, $p_{a}=0.25$, step size *α* = 1, and *λ* = 1.5
TLBO [44]	TF_max_ = 2 and TF_min_ = 1
PSO [41]	Inertia factor = 0.3, *c*_1_ = 2, and *c*_2_ = 2
GA [42]	Crossover probability = 0.8 and mutation probability = 0.05
SSA [38]	The number of leaders = *N*/2
HHO [37]	Initial state = 2

**Table 5:** Results of IHHO and nine conventional swarm-based algorithms on 23 benchmarks.

Function	Metric	IHHO	HHO	SSA	DE	WOA	ABC	CS	TLBO	PSO	GA
$f_{1}$	Average	**9.8191E-108**	1.4371E-92	1.5602E-07	7.7905E+01	2.1530E-73	1.1409E+02	7.5264E+00	2.5870E-89	1.0381E-09	4.1818E-01
	Std. Dev.	**4.4397E-107**	7.8627E-92	2.0095E-07	3.7708E+02	8.9376E-73	4.8459E+01	2.1409E+00	4.6942E-89	1.3662E-09	3.3787E-01
$f_{2}$	Average	**3.0532E-57**	2.6479E-49	1.6734E+00	6.2743E-02	3.1615E-48	5.7348E+01	1.0723E+01	3.8157E-45	2.0393E-03	4.2087E+00
	Std. Dev.	**9.1102E-57**	1.4397E-48	1.2626E+00	2.1282E-01	1.0594E-47	3.9230E+01	4.1260E+00	3.1791E-45	9.3505E-03	1.7632E+00
$f_{3}$	Average	**2.5765E-85**	2.5366E-73	1.8195E+03	6.2831E+02	4.4019E+04	6.8920E+04	2.2440E+03	1.0301E-17	1.8531E+02	1.2535E+01
	Std. Dev.	**1.4091E-84**	1.3893E-72	1.0367E+03	4.4083E+02	1.4134E+04	1.1351E+04	4.7799E+02	2.6023E-17	1.0574E+02	1.4649E+01
$f_{4}$	Average	**6.8699E-53**	2.1653E-48	1.0598E+01	2.5240E+01	5.5366E+01	6.3423E+01	9.7881E+00	1.1145E-36	2.8576E+00	1.9379E+00
	Std. Dev.	**3.4390E-52**	1.0414E-47	3.1624E+00	6.7334E+00	2.8293E+01	5.1929E+00	1.9909E+00	8.7162E-37	8.5118E-01	4.4034E-01
$f_{5}$	Average	**6.1507E-04**	1.9393E-02	2.9493E+02	1.0868E+04	2.7984E+01	2.4351E+06	4.6646E+02	2.5510E+01	4.9218E+01	1.1337E+02
	Std. Dev.	**6.4890E-04**	2.4361E-02	5.1965E+02	2.3958E+04	4.7925E-01	1.0799E+06	1.9434E+02	6.2797E-01	3.4270E+01	5.8954E+01
$f_{6}$	Average	2.6096E-06	1.8993E-04	1.5566E-07	9.1127E+00	3.7682E-01	1.1210E+02	7.5379E+00	9.0981E-05	**2.1010E-09**	1.1791E+00
	Std. Dev.	4.2167E-06	2.9303E-04	2.2753E-07	2.3285E+01	2.4227E-01	6.2740E+01	3.4105E+00	1.9284E-04	**3.7393E-09**	1.2221E+00
$f_{7}$	Average	**1.5171E-04**	1.5382E-04	1.6333E-01	6.9135E-02	2.5776E-03	8.1377E-01	7.3337E-02	1.0832E-03	1.7265E-02	1.1592E+00
	Std. Dev.	**1.2809E-04**	1.6972E-04	9.7234E-02	5.3048E-02	3.3112E-03	2.4526E-01	1.9549E-02	4.4530E-04	7.3532E-03	5.5957E-01
$f_{8}$	Average	−1.2302E+04	**−1.2554E+04**	-7.3403E+03	−7.6939E+03	−1.1026E+04	−3.1224E+03	−8.1127E+03	−7.5624E+03	−6.2607E+03	−5.3371E+02
	Std. Dev.	5.9923E+02	**3.4318E+01**	6.3915E+02	1.2408E+03	1.6428E+03	8.9440E+60	2.5378E+02	9.5457E+02	8.7230E+02	3.5126E+01
$f_{9}$	Average	**0.0000E+00**	**0.0000E+00**	4.9317E+01	1.3880E+02	3.7896E-15	2.5014E+02	1.0510E+02	1.1205E+01	3.7245E+01	2.3606E+01
	Std. Dev.	**0.0000E+00**	**0.0000E+00**	1.8591E+01	4.1840E+01	1.4422E-14	1.4647E+01	1.1449E+01	7.9283E+00	1.3498E+01	7.9342E+00
$f_{10}$	Average	**8.8818E-16**	**8.8818E-16**	2.3523E+00	1.6160E+00	4.4409E-15	3.7927E+00	2.5517E+00	6.3357E-15	5.5614E-01	1.7984E+00
	Std. Dev.	**0.0000E+00**	**0.0000E+00**	1.0343E+00	7.8250E-01	2.4685E-15	2.2979E-01	4.1469E-01	1.8027E-15	7.7138E-01	6.2565E-01
$f_{11}$	Average	**0.0000E+00**	**0.0000E+00**	1.7518E-02	2.9595E-01	**0.0000E+00**	2.1273E+00	1.0901E+00	**0.0000E+00**	1.4493E-02	2.2855E-02
	Std. Dev.	**0.0000E+00**	**0.0000E+00**	1.5344E-02	6.5631E-01	**0.0000E+00**	4.9385E-01	4.1013E-02	**0.0000E+00**	1.5665E-02	2.4988E-02
$f_{12}$	Average	**8.9331E-07**	2.8996E-06	4.8967E-01	2.7740E-02	1.1222E-02	7.0510E+00	5.5754E-01	3.4633E-03	2.0734E-02	2.0734E-01
	Std. Dev.	**1.0057E-06**	4.1578E-06	2.8196E-01	5.4624E-02	7.4349E-03	1.3382E+00	3.0349E-01	1.8926E-02	4.2177E-02	1.4924E-01
$f_{13}$	Average	**1.2617E-05**	3.0911E-05	8.8144E-02	2.0079E-02	1.4800E-01	2.9343E+00	8.3980E-02	5.2411E-02	6.2262E-03	6.9511E-02
	Std. Dev.	**1.9505E-05**	4.5579E-05	2.2500E-01	3.3155E-02	1.0076E-01	1.0096E+00	2.9587E-02	7.3284E-02	8.9788E-03	7.1892E-02
$f_{14}$	Average	**9.9800E-01**	1.8223E+00	1.4273E+00	1.1955E+00	3.2893E+00	9.9867E-01	**9.9800E-01**	**9.9800E-01**	2.8411E+00	1.2003E+01
	Std. Dev.	4.4695E-16	1.4902E+00	9.9594E-01	9.1220E-01	3.3397E+00	2.4376E-03	4.2452E-16	**0.0000E+00**	2.1717E+00	7.9773E-01
$f_{15}$	Average	**4.0478E-04**	4.1574E-04	2.8157E-03	2.0786E-03	6.1588E-04	1.2013E-03	4.2754E-04	1.0424E-03	1.7770E-03	2.6560E-03
	Std. Dev.	**1.0016E-04**	2.6449E-04	5.9575E-03	5.0218E-03	5.0040E-04	1.6644E-04	2.0271E-04	3.6537E-03	5.0656E-03	2.9745E-03
$f_{16}$	Average	**−1.0316E+00**	**−1.0316E+00**	**−1.0316E+00**	**−1.0316E+00**	**−1.0316E+00**	**−1.0316E+00**	**−1.0316E+00**	**−1.0316E+00**	**−1.0316E+00**	−1.0044E+00
	Std. Dev.	2.5551E-14	4.5761E-09	4.3473E-14	6.7752E-16	4.3849E-09	1.6930E-07	5.0499E-16	6.6486E-16	6.6486E-16	1.4901E-01
$f_{17}$	Average	**3.9789E-01**	**3.9789E-01**	**3.9789E-01**	**3.9789E-01**	**3.9789E-01**	**3.9789E-01**	**3.9789E-01**	**3.9789E-01**	**3.9789E-01**	**3.9789E-01**
	Std. Dev.	3.2210E-13	7.6822E-06	5.7514E-14	**0.0000E+00**	4.3516E-05	1.8938E-05	2.0810E-13	**0.0000E+00**	**0.0000E+00**	3.1302E-09
$f_{18}$	Average	**3.0000E+00**	**3.0000E+00**	**3.0000E+00**	**3.0000E+00**	**3.9007E+00**	**3.0000E+00**	**3.0000E+00**	**3.0000E+00**	5.7000E+00	2.2802E+01
	Std. Dev.	3.0097E-13	1.5546E-06	1.8604E-12	**1.3550E-15**	4.9332E+00	3.1730E-05	1.7200E-15	1.3650E-15	1.4789E+01	3.0031E+01
$f_{19}$	Average	**−3.8628E+00**	**−3.8582E+00**	**−3.8628E+00**	**−3.8628E+00**	**−3.8575E+00**	**−3.8628E+00**	**−3.8628E+00**	**−3.8628E+00**	**−3.8628E+00**	−3.5947E+00
	Std. Dev.	1.3697E-11	5.7063E-03	2.1958E-10	2.6962E-15	5.4941E-03	8.8844E-10	**2.4643E-15**	2.7101E-15	2.6962E-15	7.4320E-01
$f_{20}$	Average	−3.2618E+00	−3.0860E+00	−3.2187E+00	-3.2348E+00	−3.2512E+00	−3.3216E+00	**−3.3220E+00**	−3.3170E+00	−3.2625E+00	−3.2467E+00
	Std. Dev.	6.1237E-02	1.1589E-01	5.3356E-02	5.3475E-02	1.1331E-01	2.2756E-03	**1.5043E-07**	2.2115E-02	6.0463E-02	5.8274E-02
$f_{21}$	Average	**−1.0153E+01**	−5.3608E+00	−7.7297E+00	−9.9848E+00	−8.8591E+00	−9.7889E+00	−1.0153E+01	−9.7481E+00	−5.0640E+00	−5.5273E+00
	Std. Dev.	**8.8280E-11**	1.1795E+00	3.3185E+00	9.2244E-01	2.1653E+00	1.0294E+00	1.8470E-06	1.3854E+00	3.0349E+00	2.7987E+00
$f_{22}$	Average	**−1.0403E+01**	−5.2518E+00	−7.5807E+00	−1.0226E+01	−8.5132E+00	−1.0403E+01	−1.0403E+01	−9.2400E+00	−6.7222E+00	−5.7084E+00
	Std. Dev.	**7.1326E-11**	9.1813E-01	3.5764E+00	9.7043E-01	2.9663E+00	7.3059E-08	2.0020E-06	2.3712E+00	3.5629E+00	2.8233E+00
$f_{23}$	Average	**−1.0536E+01**	−5.1221E+00	−8.7358E+00	−9.8342E+00	−8.1769E+00	**−1.0536E+01**	−1.0536E+01	**−1.0536E+01**	−6.2903E+00	−4.1986E+00
	Std. Dev.	**8.7812E-11**	1.1132E-02	3.0954E+00	2.1477E+00	3.2227E+00	1.7148E-07	9.2141E-06	1.2342E-10	3.6204E+00	2.4535E+00
+/=/−		−/−/−	16/6/1	17/5/1	18/1/4	21/2/0	22/1/0	16/4/3	17/1/5	17/2/3	23/0/0
ARV		1.61	4.26	6.04	6.09	5.87	7.35	5.87	4.04	6.13	7.74
RANK		1	3	6	7	4	9	5	2	8	10

**Table 6:** *p*-values of the Wilcoxon rank-sum test comparing IHHO with conventional algorithms for all functions.

Function	HHO	SSA	DE	WOA	ABC	CS	TLBO	PSO	GA
$f_{1}$	3.8202E-10	3.0199E-11	3.0199E-11	3.0199E-11	3.0199E-11	3.0199E-11	3.0199E-11	3.0199E-11	3.0199E-11
$f_{2}$	5.5727E-10	3.0199E-11	3.0199E-11	3.0199E-11	3.0199E-11	3.0199E-11	3.0199E-11	3.0199E-11	3.0199E-11
$f_{3}$	1.6980E-08	3.0199E-11	3.0199E-11	3.0199E-11	3.0199E-11	3.0199E-11	3.0199E-11	3.0199E-11	3.0199E-11
$f_{4}$	5.4617E-09	3.0199E-11	3.0199E-11	3.0199E-11	3.0199E-11	3.0199E-11	3.0199E-11	3.0199E-11	3.0199E-11
$f_{5}$	1.8608E-06	3.0199E-11	3.0199E-11	3.0199E-11	3.0199E-11	3.0199E-11	3.0199E-11	3.0199E-11	3.0199E-11
$f_{6}$	4.1825E-09	3.4742E-10	3.0199E-11	3.0199E-11	3.0199E-11	3.0199E-11	5.3221E-03	3.0199E-11	3.0199E-11
$f_{7}$	4.6427E-01	3.0199E-11	3.0199E-11	1.4294E-08	3.0199E-11	3.0199E-11	3.3384E-11	3.0199E-11	3.0199E-11
$f_{8}$	5.3221E-03	3.0199E-11	3.0199E-11	6.5261E-07	3.0199E-11	3.0199E-11	3.0199E-11	3.0199E-11	3.0199E-11
$f_{9}$	1.0000E+00	1.2118E-12	1.2118E-12	1.6074E-01	1.2118E-12	1.2118E-12	5.7720E-11	1.2118E-12	1.2118E-12
$f_{10}$	1.0000E+00	1.2118E-12	1.2118E-12	3.6292E-09	1.2118E-12	1.2118E-12	4.6350E-13	1.2118E-12	1.2118E-12
$f_{11}$	1.0000E+00	1.2118E-12	1.2118E-12	1.0000E+00	1.2118E-12	1.2118E-12	1.0000E+00	1.2118E-12	1.2118E-12
$f_{12}$	1.2732E-02	3.0199E-11	1.6351E-05	3.0199E-11	3.0199E-11	3.0199E-11	1.0763E-02	6.7650E-05	3.0199E-11
$f_{13}$	6.8432E-01	8.1527E-11	1.4918E-06	3.0199E-11	3.0199E-11	3.0199E-11	7.6950E-08	6.6273E-01	3.0199E-11
$f_{14}$	2.9119E-10	8.8183E-01	2.7599E-09	2.1293E-11	2.1293E-11	8.3961E-01	2.9467E-12	1.4237E-03	2.1210E-11
$f_{15}$	1.0666E-07	4.5726E-09	2.3240E-02	1.2023E-08	2.4386E-09	6.7912E-01	8.5598E-04	4.8251E-01	9.9186E-11
$f_{16}$	5.4439E-11	1.3526E-01	1.1970E-12	2.9916E-11	2.9916E-11	1.3929E-10	3.2244E-12	3.2244E-12	2.9916E-11
$f_{17}$	1.0651E-05	8.9366E-01	3.4488E-07	2.6253E-11	2.6253E-11	9.1686E-01	3.4488E-07	3.4488E-07	6.4586E-11
$f_{18}$	2.0965E-08	4.2958E-08	4.0625E-12	3.0085E-11	3.0085E-11	1.6375E-09	5.1812E-12	1.0398E-09	3.0085E-11
$f_{19}$	3.0180E-11	1.7296E-02	1.7189E-12	3.0180E-11	6.0621E-11	1.4049E-11	1.2108E-12	1.7189E-12	3.0180E-11
$f_{20}$	3.0811E-08	8.5641E-04	6.9661E-02	2.9205E-02	1.0000E+00	1.0000E+00	2.2649E-07	7.4628E-04	6.1001E-01
$f_{21}$	3.0199E-11	4.5530E-01	7.7540E-11	3.0199E-11	3.0199E-11	3.8202E-10	8.5609E-07	3.8298E-04	3.0199E-11
$f_{22}$	3.0199E-11	8.8830E-01	1.9434E-10	3.0199E-11	3.0199E-11	1.6132E-10	3.3102E-04	6.6181E-01	3.0199E-11
$f_{23}$	3.0199E-11	1.2967E-01	3.9329E-08	3.0199E-11	3.0199E-11	3.0199E-11	1.4488E-11	1.8487E-01	3.0199E-11

**Table 7:** Comparison of the proposed method with existing methods.

Dataset Source	Example	Number of Points	Reported MZ	IHHO MZ	Least Square	Smaller Method ^1^	Relative Difference (%) ^2^
Huang et al. [48] (Roundness, 2021)	Example 1	25	29.2803	29.2802	29.8072	Close values	−0.000
	Example 2	24	38.2313	38.2310	39.1007	Close values	−0.001
	Example 3	100	957.413	957.420	988.236	Close values	−0.000
	Example 4	80	27.1976	27.1970	29.085	Close values	−0.002
Luo et al. [33] (Straightness, 2020)	Example 1	16	0.06693	0.06356	0.0956	IHHO MZ	−5.033
	Example 2	8	8.5200	6.3342	9.0000	IHHO MZ	−25.65
Zheng et al. [20] (Cylindricity, 2019)	Example 1	32	0.01938	0.01939	0.28558	Close values	+0.05
	Example 2	80	0.03189	0.03183	0.03661	Close values	−0.19
	Example 3	20	0.18396	0.18396	0.21197	Close values	−0.00
Radlovački V et al. [49] (Flatness, 2016)	Example 1	25	0.01840	0.01838	0.02187	Close values	−0.11
	Example 2	200	0.12252	0.12613	0.24339	Reported MZ	+2.9

^1^ “Close value” indicates an absolute relative difference between reported MZ and IHHO MZ values of less than 0.5%. ^2^ Difference between reported MZ and IHHO MZ values—relative to reported MZs (negative values indicate smaller values for IHHO).

**Table 8:** Comparison of different methods of evaluation results (unit per millimeter).

Dataset	Number of Points	Least Square	SSA	HHO	SSA&HHO	IHHO
Cylindrical surface	158	0.1627	0.4066	0.8951	0.3416	0.1013
Cylindrical axis	8	0.07509	0.06743	0.07102	0.06657	0.06610
Circular section	100	0.02898	0.02716	0.02868	0.02715	0.02715
Gauge block surface	40	0.00187	0.00184	0.00184	0.00184	0.00184

## Data Availability

Not applicable.

## Appendix A

1. Circle

The implicit expression of a circle and the corresponding distance function at a point are given in Equation (A1) and Equation (A2), respectively, where (a,b) denotes the center of the circle and *R* denotes the radius of the circle.

(A1) $${\left(X-a\right)}^{2}+{\left(Y-b\right)}^{2}-R^{2}=0$$

(A2) $$f(P_{i},U(a,b,R))=\sqrt{{\left(x_{i}-a\right)}^{2}+{\left(y_{i}-b\right)}^{2}}-R$$

2. Line

The implicit expressions of the straight line and the distance function are given in Equation (A3) and Equation (A4), respectively, where $\left(x_{0},y_{0},z_{0}\right)$ is the position and (a,b,c) is the direction vector of the spatial line.

(A3) $$\frac{X-x_{0}}{a}=\frac{Y-y_{0}}{b}=\frac{Z-z_{0}}{c}$$

(A4) $$\begin{array}{l} f(P_{i},U(a,b,c,x_{0},y_{0},z_{0}))=\\ \sqrt{\frac{{\left[b\left(x_{i}-x_{0}\right)-a\left(y_{i}-y_{0}\right)\right]}^{2}+{\left[c\left(x_{i}-x_{0}\right)-a\left(z_{i}-z_{0}\right)\right]}^{2}+{\left[c\left(y_{i}-y_{0}\right)-b\left(z_{i}-z_{0}\right)\right]}^{2}}{a^{2}+b^{2}+c^{2}}} \end{array}$$

3. Plane

The implicit expression of the plane and the corresponding distance function at a point are given in Equation (A5) and Equation (A6), respectively, where *a*, *b*, and *c* denote the normal vectors of the plane.

(A5) aX+bY+cZ+d=0

(A6) $$f(P_{i},U(a,b,c,d))=\frac{ax_{i}+by_{i}+cz_{i}+d}{\sqrt{a^{2}+b^{2}+c^{2}}}$$

4. Cylinder

The implicit expression of the cylinder and the corresponding distance function at a point are given in Equation (A7) and Equation (A8), respectively, where $\left(x_{0},y_{0},z_{0}\right)$ denotes the axis position of the cylinder, (a,b,c) denotes the direction vector of the axis, and *R* denotes the radius of the cylinder.

(A7) $$\begin{array}{l} {\left(X-x_{0}\right)}^{2}+{\left(Y-y_{0}\right)}^{2}+{\left(Z-z_{0}\right)}^{2}-R^{2}=\\ \frac{a(X-x_{0})+b(Y-y_{0})+c(Z-z_{0})}{a^{2}+b^{2}+c^{2}} \end{array}$$

(A8) $$\begin{array}{l} f(P_{i},U(a,b,c,x_{0},y_{0},z_{0},R))=\\ \sqrt{\frac{{\left[b\left(x_{i}-x_{0}\right)-a\left(y_{i}-y_{0}\right)\right]}^{2}+{\left[c\left(x_{i}-x_{0}\right)-a\left(z_{i}-z_{0}\right)\right]}^{2}+{\left[c\left(y_{i}-y_{0}\right)-b\left(z_{i}-z_{0}\right)\right]}^{2}}{a^{2}+b^{2}+c^{2}}}-R \end{array}$$
